# Cutaneous melanoma primary site is linked to nevus density

**DOI:** 10.18632/oncotarget.22016

**Published:** 2017-10-24

**Authors:** Alejandro Martin-Gorgojo, Marta Llinares, Amaya Virós, Celia Requena, Zaida Garcia-Casado, Víctor Traves, Rajiv Kumar, Eduardo Nagore

**Affiliations:** ^1^ Escuela de Doctorado, Universidad Católica de Valencia “San Vicente Mártir”, Valencia, Spain; ^2^ Department of Dermatology, Instituto Valenciano de Oncologia (IVO), Valencia, Spain; ^3^ Skin Cancer and Ageing Laboratory, CRUK Manchester Institute, Manchester, UK; ^4^ Salford Royal NHS Foundation Trust, Manchester, UK; ^5^ Department of Molecular Biology, Instituto Valenciano de Oncologia (IVO), Valencia, Spain; ^6^ Department of Pathology, Instituto Valenciano de Oncologia (IVO), Valencia, Spain; ^7^ Division of Molecular Genetic Epidemiology, German Cancer Research Center, Heidelberg, Germany; ^8^ Dermatology Department, School of Medicine, Universidad Católica de València “San Vicente Mártir”, Valencia, Spain

**Keywords:** melanoma, nevus, risk, sunburn, solar elastosis

## Abstract

There are at least two pathways driving cutaneous melanoma; one is linked to an inherent melanoma susceptibility to nevi development and the second to environmental cumulative ultraviolet light exposure. In this study, we examined the relation between nevus density, accrued sun damage and the site of primary melanoma excision.

In a series of 888 consecutive cutaneous melanoma patients, melanomas appearing in skin areas with a high relative nevus density were most prominent in men, with an elevated nevus count, at sites without solar elastosis, but with an epidemiological history of previous sunburn.

The present study associates melanoma development to sites with high nevus density. Our study supports more careful surveillance of body areas with increased nevus density in patients with high total body number of nevi, especially when they report a history of sunburns at these sites.

## INTRODUCTION

By studying risk behavior and epidemiological characteristics of melanoma patients, Whiteman *et al*. proposed a dual pathway to melanoma acquisition [1, 2]. One possible route to melanoma is the nevus-prone pathway, where intrinsic predisposition to melanocytic proliferation drives the presence of a high number of melanocytic benign and atypical nevi [3, 4]. On the other hand, a second path links the accumulation of cutaneous actinic damage throughout life, or excessive chronic or intermittent ultraviolet radiation (UVR) exposure, to melanoma [2]. Patients developing melanoma through this second pathway have typically fair skin and signs of chronic sun damage, other UVR-related non-melanoma skin cancers and the expression of p53 protein in the primary tumor [4].

Although the nevus-prone pathway underpins melanoma patients with multiple nevi, it is unclear whether the primary melanoma in this context is more likely to arise at sites where the number of nevi is highest [5–9]. Nevi are not distributed homogeneously on the body and their location can be influenced by genetic and environmental (UVR) factors, so we investigated whether the site of primary melanoma is related to the relative density of nevi on the cutaneous area where it arises. Here we present the clinical and epidemiological features of primary melanoma in relation to the nevus density at the site they develop, and investigate the care implications.

## RESULTS

Out of 2052 patients included in the database, 1550 had newly diagnosed melanomas, and 1110 individuals had sporadic invasive cutaneous melanomas, of whom a total of 888 patients had sufficient data recorded to calculate both the total nevus density and the nevus density at the melanoma region. The characteristics of the studied population are detailed in Table 1.

**Table 1:** Total population and subgroup (divided following the nevi density on the melanoma area) description, and univariate (Chi-square) analyses results

Variable	Total(N=888)		0 nevus/m^2^(N=339)		1-15 nevi/m^2^(N=238)		>15 nevi/m^2^(N=311)		P(Pc)
	N	%	N	%	N	%	N	%	
Age (mv: 0)									<0.001(<0.001)
-≤45	280	31.5	58	17.1	75	31.5	147	47.3	
-46-60	297	33.4	94	27.7	95	39.9	108	34.7	
->60	311	35.0	187	55.2	68	28.6	56	18.0	
Sex (mv: 1 [0.1%])									0.009(0.135)
-Male	433	48.8	148	43.8	112	47.1	173	55.6	
-Female	454	51.2	190	56.2	126	52.9	138	44.4	
Location (mv: 0)									<0.001(<0.001)
-Head/neck	161	18.1	101	29.8	39	16.4	21	6.8	
-Upper limb	130	14.6	48	14.2	29	12.2	53	17.0	
-Trunk	358	40.3	42	12.4	121	50.8	195	62.7	
-Lower limb	186	20.9	101	29.8	49	20.6	36	11.6	
-Acral	53	6.0	47	13.9	0	0.0	6	1.9	
Sun exposure at melanoma site (mv: 0)									<0.001(<0.001)
-None/rarely	73	8.2	51	15	9	3.8	13	4.2	
-Occasionally	640	72.1	180	53.1	181	76.1	279	89.7	
-Usually	175	19.7	108	31.9	48	20.2	19	6.1	
Number of common nevi (mv: 0)									<0.001(<0.001)
- <20	590	66.4	323	95.3	196	82.4	71	22.8	
-20-50	161	18.1	9	2.7	34	14.3	118	37.9	
-51-100	92	10.4	4	1.2	8	3.4	80	25.7	
- >100	45	5.1	3	0.9	0	0.0	42	13.5	
Total body nevi density (mv: 0)									<0.001(<0.001)
-<2.5	282	31.8	223	65.8	58	24.4	1	0.3	
-2.5-10.6	303	34.1	97	28.6	135	56.7	71	22.8	
- >10.6	303	34.1	19	5.6	45	18.9	239	76.8	
Solar lentigines (mv: 12 [1.4%])									0.198(1.0)
-No	103	11.8	43	12.9	32	13.6	28	9.1	
-Yes	773	88.2	291	87.1	203	86.4	279	90.9	
Solar lentigines on MM area (mv:6 [0.7%])									0.025(0.375)
-No	439	49.8	183	54.5	120	50.8	136	43.9	
-Yes	443	50.2	153	45.5	116	49.2	174	56.1	
Severe sunburns (mv:1 [0.1%])									0.005(0.075)
-No	407	45.9	183	54.1	105	44.1	119	38.3	
-1-5	304	34.3	99	29.3	85	35.7	120	38.6	
-6-10	69	7.8	19	5.6	22	9.2	28	9.0	
- >10	107	12.1	37	10.9	26	10.9	44	14.1	
Sunburns at MM area (mv: 10 [1.1%])									<0.001(<0.001)
-No	276	31.4	158	47.3	55	23.2	63	20.5	
-Mild	359	40.9	114	34.1	115	48.5	130	42.3	
-Intense	243	27.7	62	18.6	67	28.3	114	37.1	
Histological subtype (mv: 0)									<0.001(<0.001)
-LMM	97	10.9	67	19.8	20	8.4	10	3.2	
-SSM	581	65.4	175	51.6	174	73.1	232	74.6	
-NM	132	14.9	43	12.7	32	13.4	57	18.3	
-ALM	31	3.5	30	8.8	0	0.0	1	0.3	
-Other/NOS	47	5.3	24	7.1	12	5.0	11	3.5	
Contiguous neval remnants (mv: 32 [3.6%])									<0.001(>0.001)
-No	637	74.4	273	83.2	165	72.7	199	66.1	
-Yes	219	25.6	55	16.8	62	27.3	102	33.9	
CSD (mv:=301 [33.9%])									<0.001(<0.001)
-No	507	86.4	178	76.1	140	89.2	189	96.4	
-Yes	80	13.6	56	23.9	17	10.8	7	3.6	
Stage (mv: 0)									0.958(1.0)
-In situ	154	17.3	62	18.3	39	16.4	53	17.0	
-Local disease	617	69.5	230	67.8	170	71.4	217	69.8	
-Locoregional	115	13.0	46	13.6	29	12.2	40	12.9	
-Metastatic	2	0.2	1	0.3	0	0.0	1	0.3	
Non-synonymous									0.032
MC1R variants (mv: 0)									(0.480)
-None	280	33.8	118	38.2	73	32.6	89	30.1	
-1 variant	332	40.0	118	38.2	101	45.1	113	38.2	
->1 variant	217	26.2	73	23.6	50	22.3	94	31.8	

mv: missing values.

CSD: severe chronic sun damage on the skin surrounding the melanoma.

P: p-value from Chi squared test.

Pc: p-value corrected by Bonferroni test.

When patients were grouped according to the nevus density at the melanoma region, a high nevus density was significantly associated with young age at presentation, a high total body nevus count and density, and presence of one or more non-synonymous MC1R variants. These patients more frequently presented the melanoma on the trunk and had a history of intermittent sun exposure where the patient recalled having had sunburns in the past. The tumor was mainly of the SSM subtype and presented without histological signs of chronic sun damage (absent solar elastosis, non-CSD) (Table 1).

**Figure 1:**
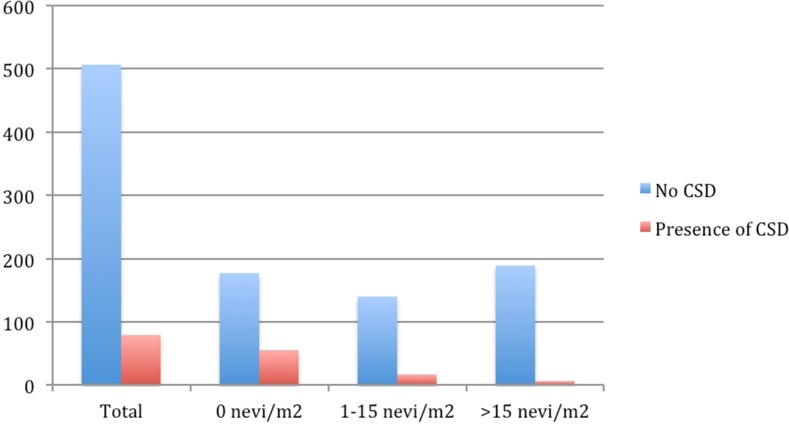
Number of patients showing histological evidence of CSD according to the nevi density on the melanoma area

When comparing patients classified by the site-specific relative nevus density, melanomas located on an area with a higher nevus density than the total body density occurred more frequently in patients who were men (53.3%), were 45 years of age or younger (39.1%) and had a high total body nevus density (49.1% had >10.6 nevi/m^2^). The frequency of patients with increased relative nevus density at melanoma site increased progressively with increase in total body nevus density; with 18.3%[50/273] in the group with <2.45 nevi/m^2^, 52.8%[160/303] in the group with 2.45-10.60 nevi/m^2^ and 69.2%[211/303] in the group with >10.60 nevi/m^2^. In addition, the SSM subtype tumors were more frequent (73.7% of patients), developed in intermittently sun-exposed areas (85% of patients), and the patients recalled having suffered sunburns. In our study 90% of patients recalled having suffered at least one sunburn at the site, with 35.7% remembering at least one severe, blistering sunburn. This epidemiological history was consistent with the presence of solar lentigines (58.2%) but absent solar elastosis with 93.5% of patients compared to 79.9% in the group with relative nevus density lower than or equal to the total body nevus density. The 32.8% of primary melanomas had an increased frequency of contiguous neval remnants compared to 18.8% in the group with low site-specific nevus density (Table 2). The age-adjusted multivariate analysis confirmed a high total body nevus density with 6.5X for third and 4.2X for second tertiles. The absence of solar elastosis at the primary melanoma site, and a past history of sunburns at the melanoma site were the relevant variables that significantly associated with the development of tumors in areas with a high relative nevus density (Table 3). Furthermore, we describe a progressive decrease in solar elastosis with increasing melanocyte count in melanoma patients (Figure 1).

**Table 2:** Characteristics of the groups defined by the relative nevi density on the melanoma area compared to the total body surface nevi density

	Nevi density on the MM area lower or equal to total body nevi density		Nevi density on the MM area higher to total body nevi density		
Variable	N	%	N	%	P(Pc)
Age (mv: 0)					<0.001
-≤45	112	24.5	168	39.1	(<0.001)
-46-60	139	30.3	158	36.7	
->60	207	45.2	104	24.2	
Sex (mv: 1 [0.1%])					0.01
-Male	204	44.6	229	53.3	(0.15)
-Female	253	55.4	201	46.7	
Location (mv: 0)					<0.001
-Head/neck	121	26.4	40	9.3	(<0.001)
-Upper limb	61	13.3	69	16.0	
-Trunk	82	17.9	276	64.2	
-Lower limb	146	31.9	40	9.3	
-Acral	48	10.5	5	1.2	
Sun exposure of melanoma site (mv: 0)					<0.001
-None/rarely	60	13.1	13	3.0	(<0.001)
-Occasionally	272	59.4	368	85.6	
-Usually	126	27.5	49	11.4	
Number of common nevi (mv: 0)					<0.001
- <20	372	81.2	218	50.7	(<0.001)
-20-50	48	10.5	113	26.3	
-51-100	22	4.8	70	16.3	
- >100	16	3.5	29	6.7	
Total body nevi density (mv: 0)					<0.001
-<2.5	223	48.7	50	13.7	(<0.001)
-2.5-10.6	143	31.2	160	37.2	
- >10.6	92	20.1	211	49.1	
Solar lentigines (mv: 12 [1.4%])					0.679
-No	55	12.2	48	11.3	(1.0)
-Yes	396	87.8	377	88.7	
Solar lentigines on MM area (mv:6 [0.7%])					<0.001
-No	260	57.3	179	41.8	(<0.001)
-Yes	194	42.7	249	58.2	
Number of severe sunburns (mv:1[0.1%])					0.123
-No	224	49.0	183	42.6	(1.0)
-1-5	151	33.0	153	35.6	
-6-10	28	6.1	41	9.5	
- >10	54	11.8	53	12.3	
Sunburns at the MM area (mv: 10 [1.1%])					<0.001
-No	195	43.1	81	10.0	(<0.001)
-Mild	166	36.7	193	45.3	
-Intense	91	20.1	152	35.7	
Histological subtype (mv: 0)					<0.001
-LMM	73	15.9	24	5.6	(<0.001)
-SSM	264	57.6	317	73.7	
-NM	61	13.3	71	16.5	
-ALM	30	6.6	1	0.2	
-Other/NOS	30	6.6	17	4.0	
Contiguous neval remnants (mv: 32 [3.6%])					<0.001
-No	358	81.2	279	67.2	(<0.001)
-Yes	83	18.8	136	32.8	
CSD (mv:=301 [33.9%])					<0.001
-No	247	79.9	260	93.5	(<0.001)
-Yes	62	20.1	18	6.5	
Stage (mv: 0)					0.986
-In situ	80	17.5	74	17.2	(1.0)
-Local disease	316	69.0	301	69.5	
-Locoregional	61	13.3	54	13.0	
-Metastatic	1	0.2	1	0.2	
Non-synonymous MC1R variants (mv: 0)					0.181
-None	155	36.7	125	30.7	(1.0)
-1 variant	160	37.9	172	42.3	
->1 variant	107	25.4	110	27.0	

P: p-value by Chi-squared test.

Pc: p-value corrected by Bonferroni test.

**Table 3:** Age-adjusted multivariate regression model for the characteristics significantly associated to a site-specific relative nevus density higher than the total nevus density

Variable	OR	95% CI	P
Female vs. male	0.5	0.4-0.8	0.002
Total body nevi density:			
-<2.5	Ref.	Ref.	
-2.5-10.6	4.2	2.6-6.8	<0.001
- >10.6	6.5	3.9-10.9	<0.001
Sunburns at melanoma area:			
-No	Ref.	Ref.	
-Mild	3.2	2.0-5.1	<0.001
-Intense	3.9	2.3-6.6	<0.001
CSD			
-No	Ref.	Ref.	0.001
-Yes	0.3	0.2-0.6	

Ref: Reference.

## DISCUSSION

In this study using data from 888 melanoma patients we describe the characteristics of melanomas according to total body nevus density and according to nevus density at the site of primary melanoma. Melanomas arising in areas with a high nevus density and the tumors developing in cutaneous areas with a site-specific relative nevus density greater than the total body nevus density are more frequently observed in men with a high total body nevus density and count. These tumors more likely arise in previously sunburned skin areas as recalled by the patient, and the peritumoral skin presents no histological signs of chronic sun damage.

Nevus density and total number of nevi have been known for decades to be an important risk factor to melanoma [10–12]. Total nevus density has been linked to sun exposure [13], lighter phototypes, a history of sunburns in children [14, 15], melanomas with contiguous neval remnants [16], familial cases of dysplastic nevus syndrome and melanoma [17], genetic cell-cycle control factors [18, 19] such as the CDKN2A gene [20, 21] and others [22], as well as an absent p53 expression within melanoma tumor samples, which supports the divergent pathway model [1, 23]. Although the association between primary tumor site and nevus distribution has been noted this has not yet been studied in depth [24–28] and, therefore, there are currently no clear recommendations regarding variations in regional anatomic or site-specific nevus density.

The present study is, to our knowledge, the first to assess subsets of patients specifically according to both absolute and site-specific relative nevus density in 888 prospectively recruited melanoma patients with inclusion of clinical, histological and genetic susceptibility factors. The data analysis has shown that patients presenting with a higher site-specific relative nevus density are more frequently male, younger, with a higher total body nevus density, a past history of sunburns on the melanoma area, and with histopathological findings showing no chronic sun damage.

Our findings link a higher nevus density at the melanoma area with younger patients, which could be associated to the progressively lower nevus count observed during ageing. Male sex has also proven to be an independent associated factor [24]. This could be due to the higher frequency of melanoma on the back and shoulders in males, areas with overall higher melanocytic nevi counts [3]. Additionally, men report different patterns of sun exposure and behavior [29]. In a similar manner, the SSM histological subtype may be associated to high nevus density due to its overall higher incidence and its well-established link to overall higher nevus counts [25]. We also found an increased frequency of contiguous neval remnants at sites of higher nevus density which could be justified by the inherent association of melanoma arising over a nevus, however this question has not been examined in detail.

Our data supports that patients with high overall nevi numbers have a significantly increased risk of presenting a melanoma in areas with a high relative nevus density [3, 25]. We hypothesize this observation could be due to either a site-specific higher melanocyte proliferation, to the influence of greater sun exposure at this area, or due to a combination of both factors. The strong link between nevus density, primary melanoma site and a prior history of sunburn or intermittent UVR exposure at the same site support the role of sun exposure as a trigger for melanoma development in an already predisposed area [30–32]. The present findings suggest that areas with a high nevus density that have suffered mild or intense sunburns should be clinically examined more carefully, and should probably need to be more meticulously sun protected in order to prevent the increase in the number of nevi, an approach which has been proven especially useful in fair-skinned, freckle-prone patients [19].

By contrast, CSD melanomas are confirmed to arise more frequently on chronically sun-exposed areas -such as the head and neck- in patients with non-melanoma skin cancers and a low number of nevi [2, 33, 34]. These data further supports the divergent etiologic pathway model [2].

Importantly, we show that the higher the nevus density at a primary melanoma site, the less likely it is to present CSD. Using CSD as a surrogate marker of cumulative sun damage, our finding strengthens the idea that individuals with increased propensity (susceptibility) to nevus development require less total sun exposure to progress to melanoma. The gradual decline in CSD as the number of nevi increase in our patients supports the idea that the two main pathways for cutaneous melanoma are not mutually exclusive but complimentary, representing two ends of an etiopathological spectrum where a higher inherent nevus propensity requires less UVR cooperation, and in individuals with lower nevus density there is progressive increased UVR involvement for melanomagenesis.

An advantage of our study is that all patient data were collected prospectively, following homogeneous criteria, assessed by the same clinicians within a national tertiary reference center with a wide geographical catchment area. Thus, our cohort is a faithful extrapolation of Spanish melanoma patients. However, certain limitations may affect this study, such as its sample size, the inherent constraints associated to a retrospective approach and the intrinsic recall bias in the history of sunburns at the site of primary melanoma incidence, although the latter affects equally all the patients who did not know the purpose of the present study when providing this information.

In summary, we show that a higher nevus density at a given area increases the risk of developing melanoma at this site, particularly in male patients who have a high overall nevus density, a past personal history of sunburns at the site of melanoma. This is progressively and inversely correlated to histological evidence of chronic sun damage. Identifying these individuals at high-risk will help tailor surveillance and monitoring campaigns in the primary care setting.

## MATERIALS AND METHODS

In this retrospective, observational study, data from the melanoma database of the Dermatology Department of the *Instituto Valenciano de Oncologia* (IVO), Valencia, Spain, were analyzed. This database, launched in 2000, has been regularly updated with data from all newly diagnosed and follow-up melanoma patients. Clinical, epidemiologic, and histological data are collected prospectively, including the medical history and physical examination of patients performed by interview by dermatologists with experience in melanoma management [29]. Specifically, nevus count was performed by an experienced dermatologist (E.N.) and included all unequivocal nevi (when appropriate this fact was assessed by dermoscopy) above 2 mm of diameter.

The present study was approved by our institution's Research Ethics Board. Informed consent was obtained from all participants.

Inclusion criteria were incident patients with invasive sporadic cutaneous melanoma, who had received definitive treatment at our institution between January 1, 2000, and December 31, 2014. Patients with extracutaneous melanomas and those with metastatic melanomas and unknown primary tumor were excluded.

The hypothesis of the study was that melanomas in patients with high number of total nevi develop mainly in areas with increased nevus density. Therefore, the primary outcome measure was the site-specific relative nevus density, which was defined as the ratio of the site specific nevus density to the total body nevus density for each person.

For the purpose of this study, we calculated the nevus density both according to overall skin surface and to the region where the melanoma had developed. Total body nevus density was calculated by dividing the number of common nevi greater than 2 mm in diameter by the body surface. This surface was defined according to the DuBois formula: (weight (kg)^0.425^) x (height (cm)^0.725^) x 0.007184- [35]. Nevus density at the melanoma region was calculated counting the number of nevi at the region where the melanoma presented and defining ‘region’ using Wallace's rule of nines to infer the proportion of total body surface: head (9% of the total body surface), upper limb (7% excluding the hand), hand (2%) anterior trunk (18%), posterior trunk (18%), lower limb (15% excluding the foot), foot (3%) [36]. Thereafter, three categories of nevus density at the melanoma area were defined by tertiles: 0 nevi/m^2^ (low density), 1-15 nevi/m^2^ (intermediate density) and >15 nevi/m^2^ (high density).

For the comparative analyses, the following variables were used:

1. Epidemiological variables: age (<45, 45-60, or >60 years), sex, melanoma location (head/neck, trunk, upper limb, lower limb, and acral locations).

2. Phenotypic traits: “photolocation” or melanoma location according to sun exposure patterns (rarely/non-exposed, occasionally exposed, usually exposed), number of common melanocytic nevi (<20, 20-50, 51-100 and >100), nevus density on the complete body surface (total number of nevi/square meters of body surface, further categorized according to tertiles into: <2.5, 2.5-10.6 and >10.6), presence of solar lentigines, and presence of solar lentigines at the melanoma area.

3. Environmental factors: past personal lifetime history of severe sunburns (none, 1-5, 6-10, >10), past personal history of sunburns at the melanoma site (none, mild, intense).

4. Histopathological criteria: melanoma subtype (superficial spreading melanoma [SSM], lentigo maligna melanoma [LMM], nodular melanoma [NM], acral lentiginous melanoma [ALM] or other/non-specified [Other/NOS]), contiguous neval remnants, solar elastosis (as a sign of cumulative sun-damage –[CSD] and categorized in CSD vs. non-CSD according to previous classification [37]), and stage (*in situ*, local disease, locorregional disease, metastatic disease). All slides were reviewed by the same pathologist to avoid interobservational bias.

3. Germline susceptibility: presence of any non-synonymous MC1R variants (Supplementary Table 1) determined by direct sequencing according to previously described methods and categorized into none, one or more than one variant [38].

We performed two independent comparisons. First, the aforementioned characteristics were compared between the three groups defined by the density of nevi within the melanoma primary site. Second, we compare the characteristics between the two groups defined by the site-specific relative nevus density: one group where the nevus density at the melanoma area was lower or equal to the total body nevus density (a ratio value less than or equal to one), and another in which it was higher (a ratio value greater than one).

The differences between the distributions of each variable in our groups were evaluated using Pearson's chi-square test. Odds ratios (OR) were calculated by univariate and age-adjusted stepwise forward multivariate logistic regression to assess the association of each variable to a site-specific relative nevus density higher than the total nevus density. All the statistical analyses were performed using the SPSS statistical package for Windows, version 20.0 (IBM Inc; Illinois, USA).

## SUPPLEMENTARY MATERIALS FIGURES AND TABLES


