# Correction: Cutaneous melanoma primary site is linked to nevus density

**DOI:** 10.18632/oncotarget.26445

**Published:** 2018-12-04

**Authors:** Alejandro Martin-Gorgojo, Marta Llinares, Amaya Virós, Celia Requena, Zaida Garcia-Casado, Víctor Traves, Rajiv Kumar, Eduardo Nagore

**Affiliations:** ^1^ Escuela de Doctorado, Universidad Católica de Valencia “San Vicente Mártir”, Valencia, Spain; ^2^ Department of Dermatology, Instituto Valenciano de Oncologia (IVO), Valencia, Spain; ^3^ Skin Cancer and Ageing Laboratory, CRUK Manchester Institute, Manchester, UK; ^4^ Salford Royal NHS Foundation Trust, Manchester, UK; ^5^ Department of Molecular Biology, Instituto Valenciano de Oncologia (IVO), Valencia, Spain; ^6^ Department of Pathology, Instituto Valenciano de Oncologia (IVO), Valencia, Spain; ^7^ Division of Molecular Genetic Epidemiology, German Cancer Research Center, Heidelberg, Germany; ^8^ Dermatology Department, School of Medicine, Universidad Católica de València “San Vicente Mártir”, Valencia, Spain

**This article has been corrected:** The correct email for correspondence author is given below:

***Correspondence to****: Eduardo Nagore,* ***email****:* eduardo.nagore@ucv.es

Original article: Oncotarget. 2017; 8:98876-98886. https://doi.org/10.18632/oncotarget.22016

