# Supplementary material for: Cutaneous melanoma primary site is linked to nevus density
Source: Oncotarget. 2017 Oct 24;8(58):98876–86. doi: 10.18632/oncotarget.22016 (PMC5716774; doi:10.18632/oncotarget.22016)
Supplement: Supplementary file 1 [file oncotarget-08-98876-s001.pdf]

# Cutaneous melanoma primary site is linked to nevus density

## SUPPLEMENTARY MATERIALS

**Supplementary Table 1: Distribution of non-synonymous MC1R variants in melanoma patients (No.=829)**

| MC1R variant | Number of patients (%) | | MAF (%) |
|---|---|---|---|
| C35Y | 1 | (0.1) | 0.1 |
| L46F | 1 | (0.1) | 0.1 |
| L48P | 1 | (0.1) | 0.1 |
| V59M | 1 | (0.1) | 0.1 |
| V60L | 285 | (34.4) | 18.9 |
| A64T | 1 | (0.1) | 0.1 |
| R67W | 1 | (0.1) | 0.1 |
| R67Q | 1 | (0.1) | 0.1 |
| S83P | 4 | (1.0) | 0.4 |
| D84E | 8 | (1.0) | 0.5 |
| V92M | 92 | (11.1) | 5.7 |
| T95M | 2 | (0.2) | 0.1 |
| M128T | 1 | (0.1) | 0.1 |
| R142H | 23 | (2.8) | 1.4 |
| R142S | 1 | (0.1) | 0.1 |
| S145F | 1 | (0.1) | 0.1 |
| A149T | 2 | (0.2) | 0.1 |
| R151C | 74 | (9.4) | 5.0 |
| Y152X | 1 | (0.5) | 0.3 |
| I155T | 29 | (3.5) | 1.9 |
| V156L | 1 | (0.1) | 0.1 |
| R160W | 65 | (7.8) | 4.0 |
| R163Q | 32 | (3.8) | 2.0 |
| S172I | 1 | (0.1) | 0.1 |
| R213W | 2 | (0.2) | 0.1 |
| L264I | 1 | (0.1) | 0.1 |
| D294H | 67 | (8.1) | 4.4 |
| C1969DelC | 2 | (0.2) | 0.1 |
